# XtracTB Assay, a *Mycobacterium tuberculosis* molecular screening test with sensitivity approaching culture

**DOI:** 10.1038/s41598-017-03930-3

**Published:** 2017-06-16

**Authors:** Jennifer L. Reed, Debby Basu, Matthew A. Butzler, Sally M. McFall

**Affiliations:** Center for Innovation in Global Health Technologies (CIGHT), Department of Biomedical Engineering, Northwestern University, Evanston, IL 60208 USA

## Abstract

Nucleic acid amplification tests are increasingly used to diagnose tuberculosis (TB) due to their speed and sensitivity compared to sputum smear microscopy. However, these tests fail to equal culture’s sensitivity with sputum smear microscopy negative specimens and therefore cannot be used to rule out TB disease. For molecular tests to match culture’s sensitivity, they must detect ≤10 genomic copies of *Mycobacterium tuberculosis* (MTB) DNA, the limit of detection of culture, process ≥1 ml of sputum ensuring sufficient number of MTB are in the reaction, and efficiently remove sputum associated inhibitors from this large sample. Here we report the preliminary characterization of XtracTB Assay, a MTB testing protocol designed for inclusion in either an integrated point-of-care platform or a high throughput automated central laboratory system. The test combines DNA sequence specific sample prep to reduce the co-extraction of qPCR inhibitors with the amplification of two MTB specific loci (IS*6110* and *senX3-regX3)* to increase test sensitivity and minimize the likelihood of false negatives. The analytical sensitivity of the XtracTB Assay was 5 genomic copies/ml of sputum rivaling that of culture. Furthermore, 142 valid test results yield clinical sensitivity of 94.9% (95% CI: 90.1–99.9) and specificity of 100% (95% CI: 90.0–100.0).

## Introduction

Tuberculosis (TB) caused by *Mycobacterium tuberculosis* complex (MTBC) species, is the deadliest infectious disease with 1.8 million people dying from TB in 2015 world-wide^1^. Accurate and rapid diagnostic tests are crucial to reducing these unacceptably high infection and mortality rates for this deadly yet treatable disease^2, 3^. In high resource settings, pulmonary TB diagnosis relies on a combined approach of clinical symptoms, chest X-ray and sputum-based laboratory testing including sputum smear microscopy (SSM), mycobacterial culture, and more recently, molecular methods^4^. This comprehensive approach is required because no single TB test can provide sufficient diagnostic sensitivity in a timely fashion to allow for direct initiation of treatment.

Mycobacterial culture is the conventional gold standard for TB diagnosis. It is highly sensitive (limit of detection ~10–100 cfu/ml)^5^ but the time-to-result is lengthy (ranging from 2–8 weeks)^6^; moreover, the sample preparation is technically challenging, contamination prone and requires a BSL-3 facility. The time-to-result of SSM is hours versus the weeks required for culture. However, the limit of detection (LOD) of the unconcentrated smear test is 100–1000 times higher than culture (10,000 cfu/ml) and it has poor specificity in settings where non-tuberculosis mycobacteria (NTM) are commonly isolated^6^.

Nucleic acid amplification tests such as the current market leader, Xpert® MTB/RIF Assay (Cepheid; Sunnyvale, CA), provide a rapid alternative to culture or SSM for sputum smear positive (SSM+) patients with a sensitivity of 98%. However, the test sensitivity with sputum smear negative (SSM−) specimens is only 67%^7^. The suboptimal sensitivity with SSM− specimens can lead to reduced test impact, as clinicians will resort to empirical treatment for Xpert-negative patients^8^ and/or may miss the diagnosis of patients with paucibacillary TB, such as children and people living with HIV. Indeed, only 57% of TB cases reported in 2014 were bacteriologically-confirmed; the remaining were treated empirically^1^. Recently, the TB community identified the highest priority diagnostic needs and defined detailed target product profiles^9^, including a sputum-based diagnostic test to replace smear microscopy, as well as a simple and low-cost triage test that can be used at the point-of-care to rule out TB infection.

**Figure 1:**
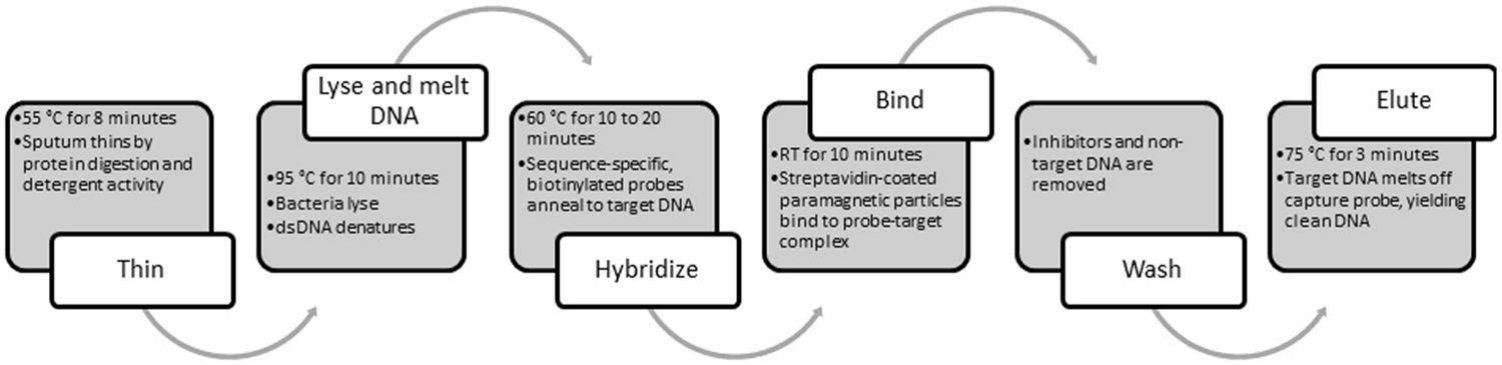
Schematic of sequence specific capture work flow: six steps, Thin, Lyse & Melt, Hybridize, Bind, Wash and Elute are indicated with corresponding times and temperatures detailed.

In order for a test to achieve the sensitivity required to rule out TB disease, it must: (1) be able to detect less than 10 genomic copies of MTB DNA (the LOD of culture), (2) process ≥1 ml of sputum to ensure that as few as 10 copies of MTB will be present in the reaction, (3) efficiently remove or evade sputum associated qPCR inhibitors from this large sample volume, and (4) specifically detect MTBC differentiating from non-tuberculosis *Mycobacterium* (NTM). Human DNA, co-extracted with MTB DNA, is a key amplification inhibitor found in sputum^10, 11^. We developed a protocol to selectively purify mycobacterial DNA utilizing a sputum thinning and MTB lysis step combined with MTB DNA sequence specific capture^11^ to circumvent this problem. One ml of sputum is thinned by enzymatic and surfactant treatment followed by a heat step that kills the bacteria and initiates the specific capture reaction by completely denaturing the DNA in the specimen (Fig. 1). MTB-specific biotinylated oligonucleotides (capture probes) are then added to the thinned sputum where a two-step capture process using streptavidin-coated paramagnetic particles (PMP) is performed. The eluted DNA is amplified using a qPCR assay that targets two MTBC-specific loci to increase sensitivity: the potentially multi-copy insertion sequence IS*6110* ^12, 13^ and the highly conserved single-copy target *senX3-regX3* that is required for virulence^14^. The limit of detection of the test was estimated to be 20 cfu/ml which is significantly lower than the Xpert® MTB/RIF assay, and no amplification was observed from a panel of 6 NTM species. In a preliminary field study of 60 de-identified blinded sputa, a test sensitivity of 96% and specificity of 100% was observed when compared to the Xpert® MTB/RIF assay^11^. The DNA capture probes and qPCR primers have been optimized for greater analytical sensitivity, and we hypothesize that this novel diagnostic test, hereby entitled XtracTB Assay, will result in improved sensitivity with SSM− specimens over current TB diagnostics.

Here, we confirm that our strategy of customizing MTB DNA extraction and amplification from 1 ml of sputum yields a LOD equivalent to culture. In a preliminary study of sputum specimens, we estimated that more than half the SSM− specimens contained less than 100 genomic copies in the qPCR reactions which is less than the Xpert®MTB/RIF Assay LOD^15^ corroborating our premise that by decreasing test LOD more SSM− samples will be detected. In a second larger study of sputum specimens, the overall clinical sensitivity of XtracTB Assay was 94.9% and specificity was 100%. Sequence specific capture, which provides DNA with low levels of qPCR inhibitors, combined with our highly sensitive qPCR assay are key elements of the XtracTB Assay demonstrated here to have the potential to replace culture in screening for pulmonary tuberculosis.

## Results

### Analytical sensitivity of XtracTB Assay is equivalent to culture

The sequence specific capture probes and qPCR oligonucleotides were optimized for Tm, and the amount of NaCl was increased from 250 mM to 500 mM to enhance sequence specific capture of MTB DNA^11^. To estimate the limit of detection, 1 ml sputum specimens were contrived to contain 20, 10 or 5 cfu/ml of MTB H37Ra which has 17 copies of IS*6110* ^16^, and 20 replicates of each concentration were assayed. None of the 8 negative sputa tested were detected by either the IS*6110* and *senX3-regX3* assays (Table 1). All 20 of the 20 cfu/ml specimens were detected by both assays. Additionally, all 10 cfu/ml specimens were detected by the IS*6110* assay, and 17 out of 20 were detected by the *senX3-regX3* assay. For the 5 cfu/ml specimens, IS*6110* assay detected 18 out of 20 (90%) and *senX3-regX3* detected 14 out of 20 (70%). The application of two diagnostic qPCR targets allowed all 20 of the 5 cfu/ml samples to be detected, surpassing the sensitivity of either single target. Therefore, the LOD of the strain H37Ra for the combined assay was estimated to be 5 cfu/ml.

**Table 1:** Limit of detection of IS*6110*, *senX3-regX3* and combined qPCR assays in the XtracTB Assay.

Genomic copies/ml	N	% Detected		
		IS*6110*	*senX3-regX3*	Combined Assay
20	20	100	100	100
10	20	100	85	100
5	20	90	70	100
0	8	0	0	0

### Similar yield of MTB DNA extracted from buffer or sputum

**Figure 2:**
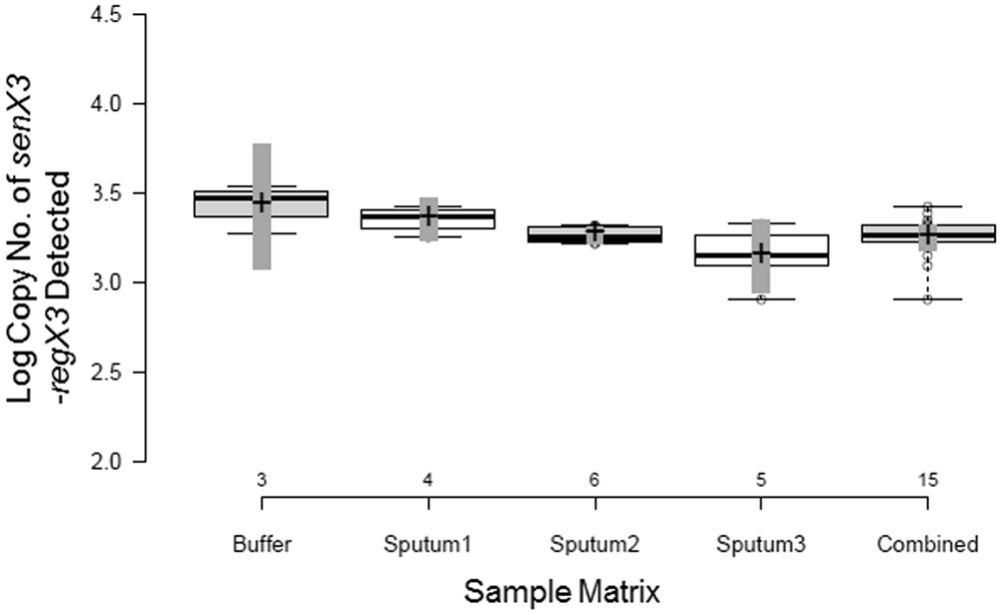
MTB DNA yield of XtracTB Assay is similar between buffer and 3 sputum specimens. Box and whisker plot of MTB yield from different specimen types: buffer, sputum 1, sputum 2, sputum 3, and all sputum results combined. Center lines show the medians; box limits indicate the 25th and 75th percentiles as determined by R software; whiskers extend to minimum and maximum values; crosses represent sample means; vertical dark gray bars indicate 95% confidence intervals of the means; data points are plotted as open circles. n = 3, 4, 6, 5, 15 sample points, respectively.

We assessed the variation of DNA yield of sequence specific extraction by testing multiple aliquots of 3 different sputum specimens and comparing the MTB DNA yield observed to spiked buffer specimens. Four to six one ml aliquots (depending upon sputum volume availability) of three residual sputum specimens were spiked with 5000 cfu/ml bacilli, and subjected to our specific capture protocol. A standard curve of MTB genomic DNA was used to calculate yields in 50% of the sample eluates as well as in a tenfold dilution of the sample eluates (Fig. 2). The yield and reproducibility of extractions as demonstrated by the coefficient of variation (CV) from each sputum specimen were very similar to that of buffer. The average yield of MTB DNA from buffer was 3.43 ± 0.14 log genomic copies/ml (95% CI: 3.08 to 3.77; CV = 4%), and the average yield of the 3 individual sputa 3.35 ± 0.07 (95% CI: 3.23 to 3.47; CV = 2%), 3.26 ± 0.04 (95% CI: 3.21 to 3.31; CV = 1%) and 3.14 ± 0.17 (95% CI: 2.94 to 3.35; CV = 5%) respectively. The results of each individual sputum specimen^15^ were combined, and their average yield was 3.25 ± 0.13 (95% CI: 3.18 to 3.32; CV = 4%). The difference in average Cq between neat eluates and tenfold dilutions (ΔCq) revealed no difference in inhibition between samples processed in buffer (ΔCq = 3.87 ± 0.06) and samples processed in sputum (ΔCq = 3.84 ± 0.22) with a p value of 0.71; an inhibited sample would have had a ΔCq of less than 3.

### Assessment of XtracTB Assay using clinical specimens: Study One

**Figure 3:**
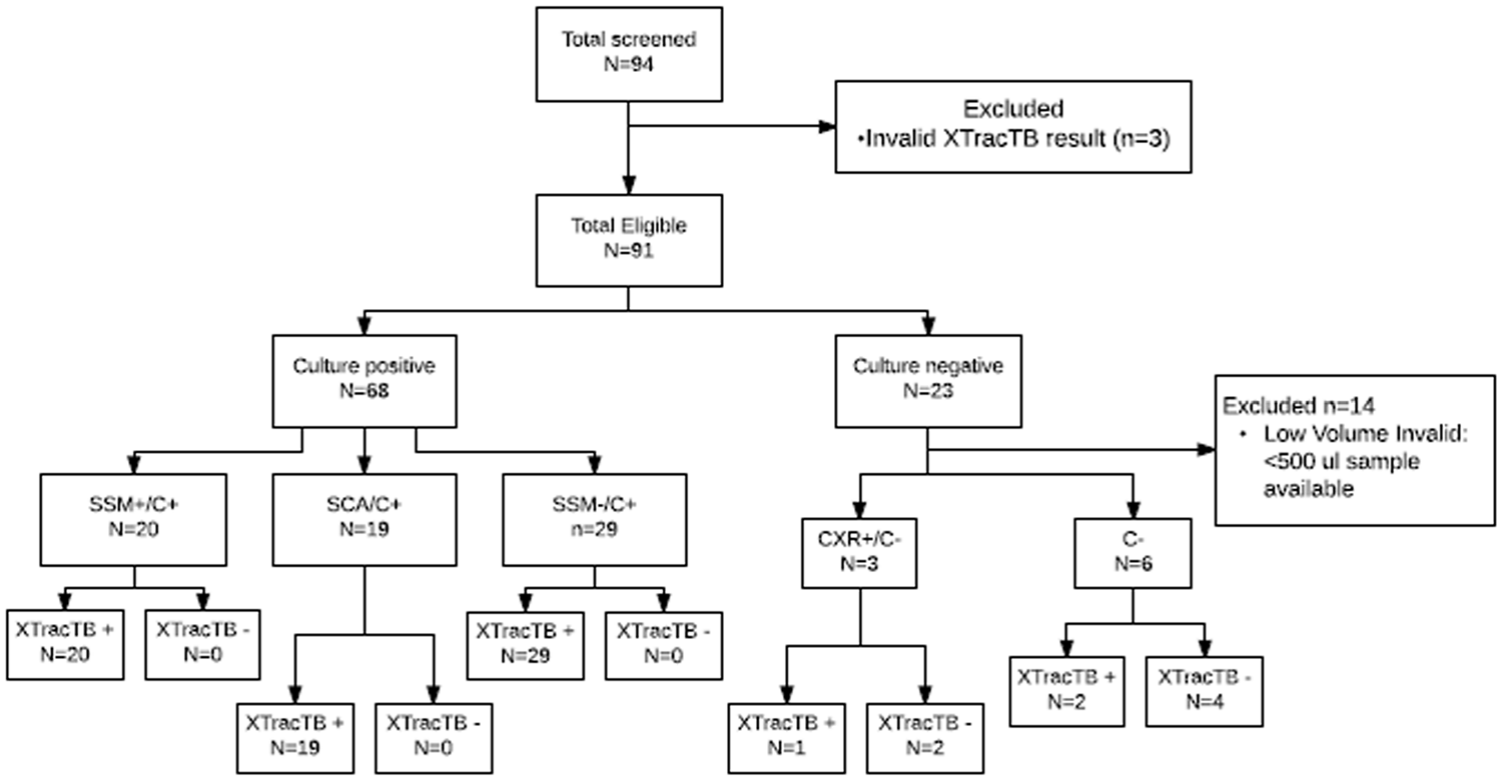
Schematic of study one protocol for sample testing. SSM− = concentrated sputum smear microscopy negative; SSM+ = concentrated sputum smear microscopy positive; C+ = culture positive; C− = culture negative; CXR+ = chest X-ray positive.

Two 500 µl aliquots of 94 independent clinical sputum specimens acquired from the Foundation for Innovative New Diagnostics (FIND) (Geneva, Switzerland) Tuberculosis Bank were combined and 950 µl was tested in the XtracTB Assay (Fig. 3). Fifty-nine of the samples were from males and 35 were from females. Fifty-two of the specimens were collected in Peru, 22 in Vietnam and 20 in South Africa. Eighty four were HIV negative, 9 were HIV positive and there was no data for one of the patients. Twenty-nine of the specimens had lower volumes than the expected 1 ml (Supplemental Tables 1 and 2). Sample volume per specimen was normalized to 950 µl with sterile water prior to testing. Samples that tested positive for either the IS*6110* or *senx3-regX3* assays were considered positive. Samples that were negative for both MTB assays and were positive for the process control (PRC) were considered negative. Samples that tested negative for both MTB assays and had a failed PRC were considered invalid.

Following sample processing and data analysis, results of clinical and microbiological testing (culture and concentrated smear) for the panel were unblinded. Three of the 94 specimens (3%) were considered invalid because both MTB targets and process control failed to amplify and were excluded from further analysis (one sputum smear negative/culture positive, one scanty smear positive & one chest x-ray positive). Of the 91 valid specimens, 68 were positive by mycobacterial culture (29 smear-negative, 19 scanty smear-positive, 20 smear-positive). All 68 of these specimens were detected by the XtracTB Assay yielding 100% sensitivity (95% CI: 93.3 to 100): 68 were positive for IS*6110* and 66 were positive for *senX3-regX3* (Table 2; Supplemental Table 1).

**Table 2:** Tuberculosis detection by XtracTB Assay of FIND culture positive specimen panel (N = 70). SSM− = concentrated sputum smear microscopy negative; SSM+ = concentrated sputum smear microscopy positive. 95% CI = 95% confidence interval. 3% of the specimens had invalid results.

Category	Reference Positive	XtracTB Positive	% Sensitivity (95% CI)
SSM−/Culture+	29	29	100 (85.4–100)
SSM+/Culture+	39	39	100 (88.8–100)
Total Culture+	68	68	100 (93.3–100)

Thirty-six of the culture positive specimens with valid XtracTB results (16 SSM− and 20 SSM+) had Xpert® MTB/RIF Assay result reported in FIND database. Of the 16 SSM− specimens, the Xpert test detected 12 (75.9%; 95% CI 47.4 to 91.7%) and XtracTB Assay detected 16 (100% 95% CI 75.9 to 100.0%). Of the 20 SSM+ specimens both Xpert® MTB/RIF Assay and XtracTB Assays detected 100% (95% CI 80.0 to 100.0%). Overall, the Xpert test detected 32 out of 36 (88.0%; 95% CI 73.0 to 96.4%) and the XtracTB test detected 36 out of 36 (100%; 95% CI 88.0–100.0%).

Of the 29 specimens with reduced volumes, 19 of them were from the culture negative sample pool (Supplemental Tables 1, and 2) which makes up 95% of the culture negative specimens tested. In fact, 60% of the culture negative specimens had volumes less than 50% of the 950 µl input volume. Because of the large number of specimens with low volume, this data set could not be used to establish test specificity. However, two of these culture negative specimens (75 and 89; Supplemental Table 1) were detected in both the IS*6110* and *senX3-regX3* assays. Eighteen of the culture negative specimens had Xpert® MTB/RIF Assay results reported in FIND database. None of the 18 was detected by the Xpert® MTB/RIF Assay (100% specificity; 95% CI 78.1 to 100.0%); this data set included the specimens 75 and 89 that were positive in the XtracTB test. Clinical notes indicated that neither of these patients had a history of TB. The chest x-ray results of one of the patients indicated pneumonia or atypical TB, but there was no other clinical or microbiological evidence supporting TB infection in these two patients. One of the samples (75; Supplemental Table 1) was a moderately strong positive, and DNA sequencing of the qPCR reaction demonstrated the presence of both the IS*6110* and the *senX3-regX3* amplicons. Sample 89 was a very weak positive, and only the IS*6110* amplicon was detected by DNA sequencing. Taken together, these results indicate that MTB DNA was present in the samples. Three further culture-negative specimens, in which chest x-ray was positive for TB and the patients had been empirically treated for TB, were screened, and one was a very weak positive in the *senX3-regX3* assay only.

### Quantification of MTB DNA in FIND panel

**Figure 4:**
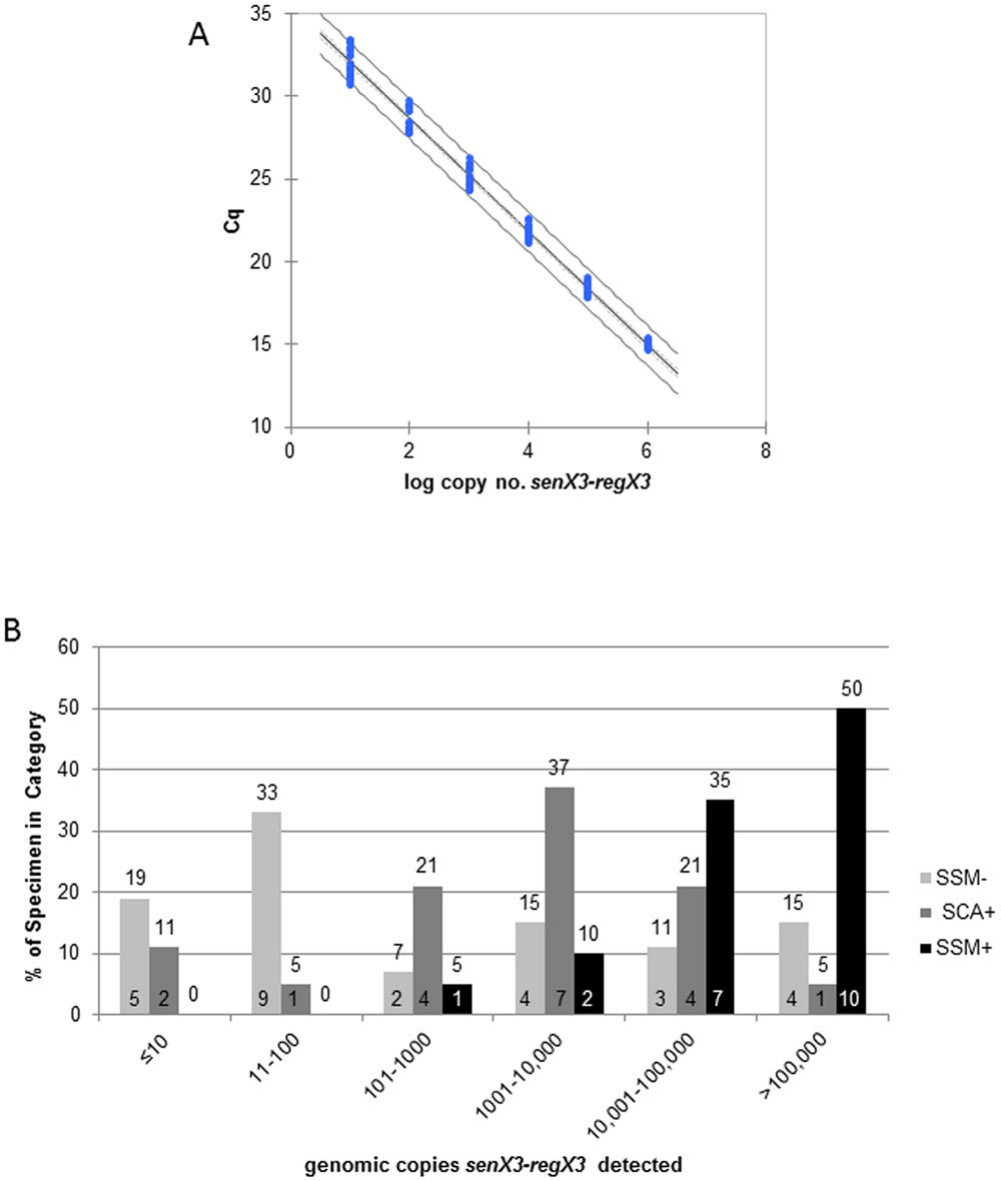
Distribution of genomic copies extracted from smear positive, scanty smear positive and smear negative samples. (**A**) Standard curve of *senX3-regX3 *log copy number 10^6^–10 copies of 7 extractions combined (118 data points). Y = −3.42X + 35.47. (95% CI of slope: −3.35, −3.48) R^2^ = 0.989. (**B**) Percentage of samples in different copy number distribution. SSM− = sputum smear microscopy negative (N = 27); SCA = scanty sputum smear positive (N = 19); SSM+ = sputum smear microscopy positive (N = 20). Numbers at top of bars indicate percentage of positive specimen in each category. Numbers at bottom of bar indicate number of positive specimen in each category.

The *senX3-regX3* Cqs were used to estimate the number of genomic copies in the qPCR reactions from the different culture positive categories: sputum smear negative (SSM−), scanty sputum smear positive (SCA) and sputum smear positive (SSM+) (Fig. 4). The IS*6110* results were not used to quantify the genomic copies detected because the number of copies of IS*6110* can vary in the MTB clinical specimens^12^. The specimen panel was tested on 7 different days, and on each day a standard curve of H37Rv genomic DNA was performed in triplicate. The Cq values of the standard curves were combined, and linear regression analysis was performed. The equation of the line was Y = −3.42X + 35.47 (95% CI of slope −3.39 to −3.45) R^2^ = 0.99. PCR efficiency was calculated to be 96.1% demonstrating that the standard curve was highly reproducible over the 7 days of the experiment. We then calculated the log copy numbers from the Cqs of the 66 *senX3-regX3* positive samples and grouped the results in categories of 10-fold dilutions (Fig. 4).

As would be expected, 95% of the SSM+ specimens were estimated to have greater than 1000 genomic copies detected per reaction with 85% of the specimens containing greater than 10,000 genomic copies. In the SCA specimen group, 79% were estimated to have between 100 and 100,000 copies per reaction but both high copy and low copy SCA samples were also detected. The SSM− population of sputum has historically been the most challenging to diagnose by molecular techniques. Of this specimen type, 52% (14/27) were estimated to have less than 100 genomic copies per reaction. However, 41% of the samples (11/27) were estimated to have greater than 1000 genomic copies of *senX3-regX3* per reaction and 25% of these specimens had more than 10,000 copies per reaction. The wide range of genomic copies observed in SSM− specimens may have resulted from the inherent biological variability of sputum specimens or the variability in performance of this challenging technique. Blakemore *et al*.^17^ also reported a substantial overlap of Cq values between smear grades indicating variability in genetic target concentration using the Xpert®MTB/RIF test and that a significant number of SSM− specimens contain relatively high numbers of MTB bacteria in their sputum.

### Performance of B. atrophaeus endospores as process control

Of the 94 sputum specimens tested, 22 (23%) were negative in the *B. atrophaeus cotJC* process control. Nineteen of these specimens were positive in at least one MTB target region with a median of 1,370 copies/sample for *senX3-regX3*. The remaining 3 *cotJC* negative specimens were classified as invalid because the two MTB targets were also negative. Furthermore, 14% of negative extraction controls performed with each run which contained buffer spiked with spores alone also failed to detect the *cotJC* amplicon. These data suggested a problem with detection of the process control alone, rather than assay inhibition that would ostensibly affect detection of all three targets processed together and not be a factor in the buffer controls. Although the sample processing, extraction, and PCR conditions were optimal for processing clinical TB-suspect sputa with high clinical sensitivity, this same method was not sufficient for processing *B. atrophaeus* spores for use as an internal extraction control. Because the results of the process control are suspect, we also calculated the clinical sensitivity including the two invalid samples which yielded clinical sensitivity for SSM− = 97% (29/30; 95% CI: 80.0–99.8), SSM+ =98% (39/40; 95% CI: 85.3–99.8) and combined culture positive = 97% (68/70; 95% CI: 8 9.1–99.5).

**Table 3:** Summary of testing results of XtracTB Assay with improved process of FIND TB specimen panel (Included specimens = 142). SSM− = concentrated sputum smear microscopy negative; SSM+ = concentrated sputum smear microscopy positive. C+ = culture positive; C− = culture negative. 95% CI = 95% confidence interval. 5% of the specimens had invalid results.

Category	Reference positive	XtracTB positive	% Sensitivity (95% CI)
SSM−/C+	35	31	88.6 (72.3–96.3)
SSM+/C+	62	61	98.4 (90.1–99.9)
Total C+	98*	93	94.9 (87.9–98.1)
	**Reference negative**	**XtracTB negative**	**% Specificity (95% CI)**
C−	44	44	100 (90.0–100)

*Note that smear data is missing for one culture-positive specimen.

### Bacillus spores are inefficiently lysed by heat step

**Figure 5:**
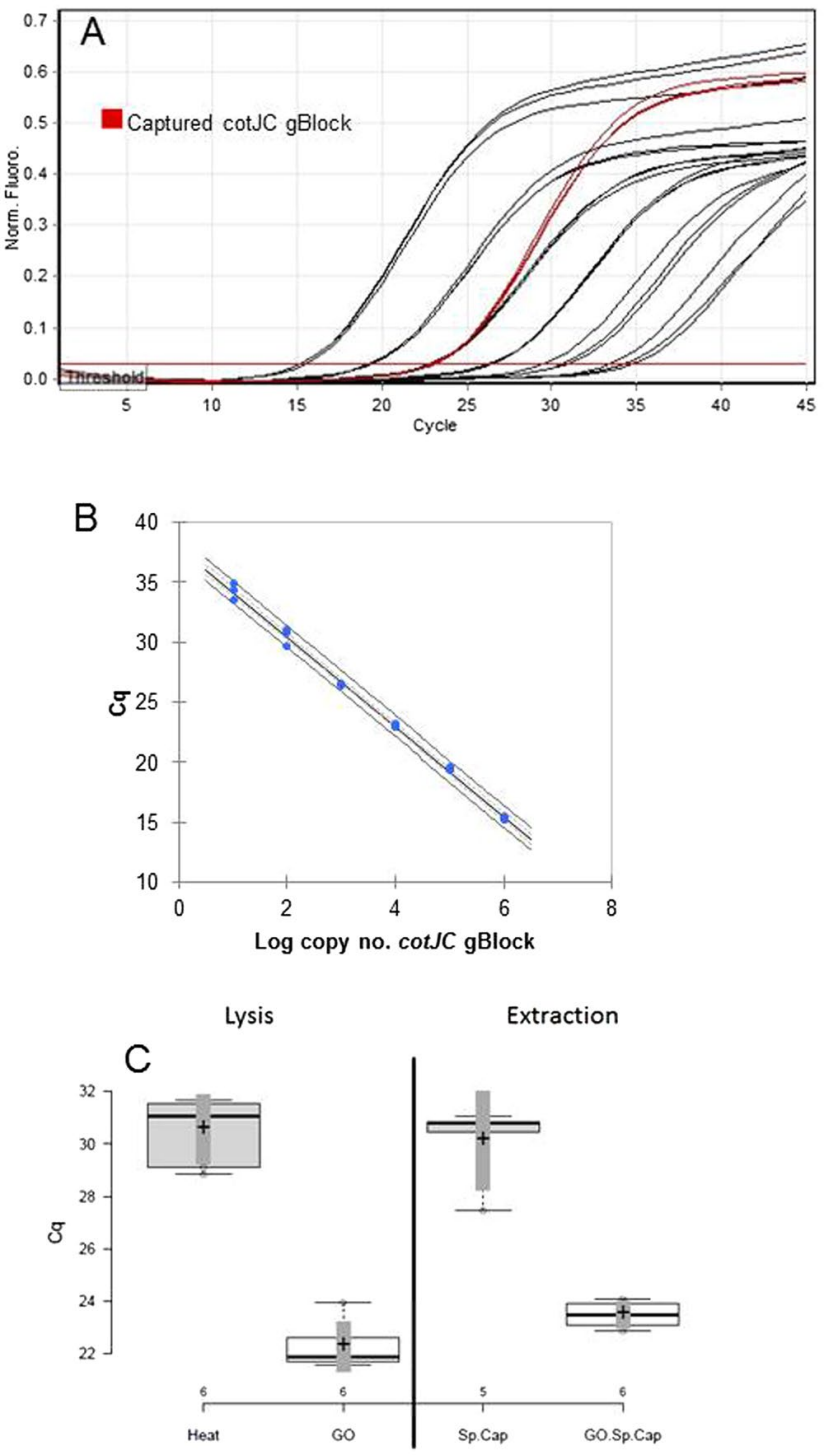
*Bacillus* spores are inefficiently lysed by heat step. (**A**) Standard curve of 1,000,000, 100,000, 10,000, 1,000, 100 and 10 *cotJC* gBlock copies in triplicate. Red curves are extracted DNA in triplicate. (**B**) *cotJC* qPCR assay is linear. Equation of line: y = −3.74x + 37.96; (95% CI slope −3.86, −3.62) R^2^ = 0.996, qPCR efficiency = 85.1%. (**C**) Box and whisker plot demonstrating that sample prep method did not sufficiently liberate *B. atrophaeus* spore DNA. Center lines show the medians; box limits indicate the 25th and 75th percentiles as determined by R software; whiskers extend to minimum and maximum values; crosses represent sample means; vertical dark gray bars indicate 95% confidence intervals of the means; data points are plotted as open circles. Samples indicated by: Heat = heat lysis; GO = GeneOhm kit; Sp.Cap = specific capture protocol; GO.Sp.Cap = GeneOhm lysis followed by specific capture protocol. n = 6, 6, 5, and 6 sample points, respectively.

The inconsistent results of the process control assay could have been the result of poor performance of the qPCR assay, inconsistent capture of the target, or inefficient lysis of the spores. Standard curve of 10–1,000,000 copies of *cotJC* gBlock® demonstrates that assay is sensitive and linear (Fig. 5A and B), and capture of 10,000 copies of *cotJC* gBlock® yielded a mean of 9385 ± 411 copies (94%) (Fig. 5A) demonstrating that both the assay and the specific capture based extraction performed as anticipated.

The lysis step in the sequence specific capture protocol involves heating specimens in the heater/shaker set at 100 °C for 10 minutes. To determine if this was sufficient to lyse *B. atrophaeus* endospores, this strategy was compared to the BD GeneOhm Lysis Kit which has the reported lysis efficiency of *Bacillus* endospores of 98.8% (package insert http://www.bd.com/ds/productCenter/441243.asp). The GeneOhm kit yielded an average Cq of 22.3 ± 0.9 compared to an average Cq of 30.5 ± 1.2 for heat alone (Fig. 5C). Two-tailed Student t test indicated that the means were significantly different (p = 1.2e-7.) These lysed samples were also subjected to DNA extraction via specific capture yielding an average Cq 23.5 ± 0.5 for the GeneOhm lysed samples and 30.1 ± 1.5 for the standard sample preparation treatment, and the Cq means were significantly different (p = 1.4e-6) The Cq differences between the GeneOhm kit lysed spores and the heat lysed spores indicates that the GeneOhm kit lyses ~200 times more spores than heating for 10 minutes at 100 °C. Therefore, we concluded that the 95 °C lysis step used in the sequence specific capture extraction protocol was not sufficient to efficiently release genomic DNA from *Bacillus* endospores and that a revamped process control was necessary to monitor the lysis of pathogens during the nucleic acid purification procedure. The DNA that we were able to detect in our previous studies was most likely free DNA from bacterial cell lysate becoming associated with the outside of spores^18^.

### Revamped process control introduced

Using traditional lambda phage cloning techniques, we generated phage particles that express the *cotJC* amplicon from *B. atrophaeus* (used above) and therefore we were able to continue to use the same multiplexed MTB qPCR assay. One thousand lambda phage particles acting as a control for cell lysis, nucleic acid extraction and qPCR amplification^19^ were added to sputum containing a range of MTB DNA from 0 to 50,000 genomic copies/ml. The average Cq of the *cotJC* assay was 27.4 ± 0.3 (range 26.9–28.2) across the range of MTB concentrations used indicating consistent performance.

### Assessment of revamped XtracTB Assay using clinical specimens: Study Two

**Figure 6:**
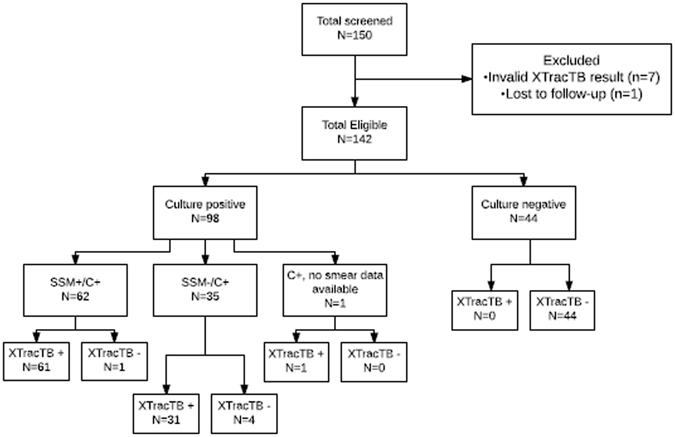
Schematic of study two protocol for sample testing. SSM− = concentrated sputum smear microscopy negative; SSM+ = concentrated sputum smear microscopy positive; C+ = culture positive.

To validate this new assay design and to establish test specificity, a second panel of 150 MTB specimens from FIND was tested (Fig. 6) using a slightly modified extraction protocol to shorten the DNA hybridization time from 20 minutes to 10 minutes. Seven of the samples were invalid in the XtracTB test due to negative TB and PRC results (5%) and one sample was eliminated by FIND due to loss to follow-up leaving 142 samples with valid test results. Eighty-six of the samples were from males and 56 were from females. One hundred and twenty four of the specimens were collected in Peru, 14 in Vietnam, and 4 in South Africa. One hundred and twelve were HIV negative, 28 were HIV positive and there was no HIV data for 2 of the patients. Only 6 of the specimens had associated Xpert® MTB/RIF Assay results. Twenty of the specimens had lower volumes than the expected 1 ml (Supplemental Table 3). Sample volume per specimen was normalized to 950 µl with sterile water prior to testing. Following sample processing and data analysis, results of clinical and microbiological testing for the panel were unblinded.

Of the 142 valid specimens, 98 were positive by mycobacterial culture (35 SSM−, 62 SSM+, and one with unknown smear status), and 44 were culture negative. Sixty-one of the 62 SSM+ specimens were detected with XtracTB assay yielding a clinical sensitivity of 98.4% (95% CI: 90.1–99.9), and 31 out of 35 of the SSM− were detected (88.6%; 95% CI: 72.3–96.3) (Table 3). The overall sensitivity of the test was 94.9% (95% CI: 87.9–98.1). None of the 44 culture negative specimens were detected by XtracTB for a clinical specificity of 100% (95% CI: 90.0–100).

## Discussion

In this study, we have demonstrated that combining sequence specific DNA extraction with multi-target MTB detection in the XtracTB Assay yields an MTB screening test with analytical and clinical sensitivity approaching mycobacterial culture. Biological factors in sputum can be inhibitory to PCR decreasing the analytical sensitivity of an MTB-specific assay by 5-fold as compared to extraction from buffer^20^. Sputum is one of the most difficult clinical specimen types as evidenced by the greater proportion of inhibited samples compared to non-pulmonary sample types when tested by Xpert® MTB/RIF (6% vs. 1%)^21^. We recently reported that a key qPCR inhibitor in sputum is the co-extraction of human genomic DNA and confirmed that the use of DNA sequence specific capture to extract the MTB DNA greatly reduces the co-extraction of human genomic DNA^10, 11^. In this study, 3 large sputum specimens were divided into 15 one ml aliquots and spiked with MTB. Intra- and inter-sample reproducibility was very high, and the MTB DNA yield extracted from the sputum specimens was very similar to the yield extracted from spiked buffer indicating that qPCR inhibitors were below assay interference levels (Fig. 2).

The analytical sensitivity of the XtracTB Assay was determined by spiking the MTB H37Ra strain, which has 17 copies of *IS6110* ^16^, in known concentrations into TB-negative sputum. The LOD was demonstrated to be 10 cfu/ml for the high copy target IS*6110*, 20 cfu/ml for the single copy *senX3-regX3* target, and 5 cfu/ml for the combined assay (Table 1). These values correspond to an LOD range that, depending upon the number of copies of IS*6110* the strain contains, is 6–26 times more sensitive than the reported LOD of the Xpert® MTB/RIF Assay, 131 cfu/ml^15^. This high analytical sensitivity was a strong predictor for the high clinical sensitivity observed. Of the 68 culture positive specimens in the first FIND panel, 68 were detected by the XtracTB Assay yielding a clinical sensitivity of 100%. In the second FIND panel, of the 98 valid culture positive specimens, 93 were detected yielding a clinical sensitivity of 95%. Unlike Xpert® MTB/RIF which utilizes a nested PCR strategy to achieve high sensitivity with SSM+ specimens (98%) but a much lower sensitivity with SSM− specimens (67%)^7^, the XtracTB Assay utilizes only one round of amplification and achieves sensitivity approaching that of culture with both SSM+ (98%) and SSM− specimens (89%).

The sample prep methods used in the two studies using clinical specimens from FIND were not identical. In the second study, we increased the amount of SDS in the sputum thinning buffer from 1% to 2%, and we also changed the composition and concentration of the salts and decreased the time of the oligonucleotide hybridization step. All these changes had been vetted in contrived sputum specimens. However, this study was the first time these conditions had been tested with clinical specimens. There was a higher invalid rate in the second study (5% vs. 3%) and a lower detection rate of SSM− specimens (89% vs. 100%). When this test is adapted to an automated system, the performance should be rigorously tested with clinical specimens.

The use of the potentially multicopy IS*6110* as a PCR target improves the sensitivity of the assay over a single copy gene. IS*6110* copy number varies from 0 to 25 in strains. Strains that lack IS*6110* are found more often in South-East Asia (8–11%) which undermines the effectiveness of this marker when testing in that region^12, 22^. Specimens from the 2 panels tested in this study were from Peru, South Africa, and Vietnam. In the first FIND panel, the SSM− strains were collected in Peru and 29/29 (100%) of valid results detected IS*6110* and 27/29 (93%) detected *senX3-regX3*. In the second FIND Panel, 20 of the SSM- samples were collected in Peru, 15 in Vietnam, and 1 in South Africa. Thirty-one out of thirty-five of the valid SSM− specimens were detected (89%). Of the 4 false negative samples, 3 of the specimens were from Viet Nam and 1 was from Peru. None of the positive tests detected *senX3-regX3* and not IS*6110*, and it is not known if any of the false negative specimens had low or no IS*6110* copies. Future studies that include more specimens from South-East Asia may allow us to determine the impact of multiple TB targets in strains that have few or no IS*6110* targets.

Culture diagnosis is the current gold standard for pulmonary tuberculosis diagnosis, but it is well recognized as an imperfect reference standard. There are multiple sources of variability in the processing of sputum for culture including: the duration of decontamination with alkali, quality of liquefaction reagent that must be prepared each day, washing conditions during centrifugation, and operator variability in centrifugation and washing steps which leads to a single culture having a reported sensitivity of only 80–85%^6^. A number of molecular tests for infectious diseases have surpassed the sensitivity of their respective culture tests^23^, and recently there have been reports of a new generation of molecular TB diagnostic tests in the pipeline that have sensitivities that approach or surpass culture^11, 24^. By combining sequence specific extraction with multicopy MTB detection, we have developed a test that is equivalent to culture in analytical sensitivity and approaches culture’s sensitivity with clinical samples (94.9%). However, as tests become more sensitive, test specificity may suffer. In our first study with FIND clinical specimens, two of the culture negative specimens were positive in both the IS*6110* and *senX3-regX3* assays, and one of the chest x-ray positive/culture negative was positive in the *senX3-regX3* assay only. In the second panel, none of the culture negative specimens were detected (100% specificity). DNA sequencing of false positive amplicons confirmed the presence of MTB in the two culture-negative sputum specimens, confirming our test detected the presence of MTB DNA in the specimens. However, the significance of the presence of the MTB DNA to TB disease is not clear.

MTB DNA detected in culture negative specimens may be the result of either culture false negatives or molecular test false positives. False negative cultures may be caused by overly harsh sputum decontamination, or the specimens may contain non-culturable bacilli due to very low growth rate for example^25^. Molecular false positive tests may be caused by residual dead bacteria or remnant DNA from a previously treated infection. It was recently reported that in high TB incidence settings, 1 out of 7 patients who have been previously treated for TB, had a false positive Xpert® MTB/RIF result relative to mycobacterial culture^26^. False positive rates are correlated with previous infections from the last 3 years^9^. Neither of the patients that had false positive results in this study reported a history of TB infection. As TB diagnostic tests become more sensitive, it may be that additional algorithms that determine if a patient has been treated for TB in the past 3 years will need to be developed to help sort out true weak positives from false positives resulting from previous infections^9, 26^.

This study has several limitations: although the sample prep method has been developed for later automation, it is currently a manual process, and it requires a highly skilled technician; our method has not been validated for quantification of MTB in specimens, and therefore, the reported bacterial loads must be considered estimates; and our current assay does not allow for detection of drug resistant TB. However, it is evident that the sample prep and amplification strategies presented here may be applied to any genetic target. As the primary diagnostic need is for a rapid, sensitive and low-cost triage test, this was the goal of our first assay development project; this work has established a proof of concept with the development of drug sensitivity tests to follow.

To validate our method for use in quantitation, we would need access to rigorously characterized reference materials as described by Devonshire, *et al*.^27^, and this study has not been done as yet. The fact that 95% of SSM+ specimens contained greater than 1000 genomic copies of *senX3-regX3* is consistent with the LOD of concentrated smear microscopy^6^ provides some internal verification to this study. SSM− specimens were observed across a range of very low concentrations of *senX3-regX3* to very high ones. The high copy number samples missed in the smear microscopy test may have resulted from the inherent biological variability of sputum specimens, the variability between specimens provided by same patients, and the fact that the smear test is a challenging technique and variables besides bacterial load play a role in determining if a sample is detected or not^28^.

DNA quantification could play an important role in TB clinical management in determining the severity of disease, risk of transmission, and response to therapy^17^. The standard curve produced by combining 7 different qPCR runs had very little variability in the slope or y-intercept, and very similar yields were extracted from 3 different sputa a total of 15 different times with minimal variation. This high reproducibility and reduction of qPCR inhibition are promising characteristics of our test that are necessary elements for a quantitative diagnostic test^11^.

This study presents preliminary analytical and clinical results indicating that by utilizing novel methods of sputum pre-treatment, DNA extraction and amplification to address the challenges of MTB diagnosis, the XtracTB Assay can approach the clinical sensitivity of culture for both SSM+ and SSM− specimens. We carefully crafted the sample prep steps for simple porting to either a high throughput automated extraction system or an integrated extraction and amplification device. The sputum pre-treatment method liquefies a large sputum volume (≥1 ml) and lyses cells without the dilution associated with liquid solutions, and the use of paramagnetic particles is amenable to direct automation. Our test could be used in high throughput automated systems in central lab settings, a small to mid-sized batch machine in a regional hospital setting and/or an integrated molecular device located near patient to both rule in and rule out a TB diagnosis. Improved molecular test sensitivity will increase the access to treatment for patients with paucibacillary TB, such as children and people living with HIV, and potentially lowering the levels of empiric treatment in test-negative patients.

## Materials and Methods

### Bacterial strains, genomic and plasmid DNA and sample sources

*Mycobacterium tuberculosis* H37Ra was acquired from the American Type Culture Collection (ATCC; Manassas, VA), and sonicated to break up cell clumps prior to contriving sputum specimens as described by Helb, *et al*.^15^. *Bacillus atrophaeus* spores (MesaLabs; Lakewood, CO, USA) were used as the process control. Residual sputa used in precision and LOD studies were obtained from TriCore Reference Laboratories (Santa Fe, NM). The recipe for synthetic sputum was adapted from Du *et al*.^29^ and consisted of 10 mg/ml porcine mucin (Sigma Aldrich, St Louis MO, USA), 1 mg/ml salmon sperm DNA (Sigma Aldrich), 3.6 mg/ml phosphatidylcholine (Sigma Aldrich), 33 mg/ml bovine serum albumin (Sigma Aldrich), and 114 mM NaCl (Sigma Aldrich).

This report includes two studies that used clinical sputum samples collected from 94 and 150 adults showing symptoms of pulmonary TB at participating clinics that were donated to the Foundation for Innovative New Diagnostics (FIND) Tuberculosis Specimen Bank. Although these repository samples are linked to clinical and microbiological information available from FIND, the specimens were de-identified, and TB status (as reflected by concentrated smear microscopy, culture, chest x-ray, clinical symptoms and MTB/RIF Gene Xpert Assay) was blinded from sample processors.

All studies presented here were performed in a research laboratory designed for real-time PCR assays so that DNA extraction, PCR master mix preparation, and DNA amplification are performed in separate rooms. Negative controls were performed with every study to monitor for potential qPCR contamination.

### Cloning of lambda phage process control

Synthetic DNA fragments (gBlocks) containing a portion of the *cotJC* gene of *Bacillus atrophaeus* were ordered from Integrated DNA Technologies (IDT, Coralville, IA, USA), and were amplified using primers with EcoRI restriction sites at the 5′ end of each primer (F primer: 5′-TTT TTG AAT TCT CAA TCA GCC ATT GGT AGG TC-3′; R primer: 5′-TTT TTG AAT TCA GCT GCA ATA TCC TGT AAA GGT C -3′) and ligated into Lambda Zap II vector predigested with EcoRI (Agilent, Santa Clara, CA, USA) using T4 DNA ligase (New England Biolabs, Ipswich MA, USA) following manufacturer’s instructions. Ligated vector was packaged into phages using Gigapack II Plus Packaging Extract (Agilent) following manufacturer’s instructions. Recombinant phages were identified by infecting host *E. coli* XLR-Blue MRF’ cells with packaged product in top agar in the presence of 5-bromo-4-chloro-3-indolyl-β-D-galactopyranoside (X-gal, Sigma Aldrich) and isopropyl-β-D-thiogalactopyranoside (IPTG, Thermo Scientific, Waltham MA, USA) as described by manufacturer. Phage containing the insert generated colorless plaques, while background plaques were blue.

Recombinant plaques were selected as follows: a clean pipette tip was inserted into the center of a colorless plaque and was rinsed in 200 ul of 0.5X SM buffer (G Biosciences, St Louis, MO, USA). 20 ul of chloroform was added, mixed, and solution was allowed to incubate at room temperature for 2 hours. The presence of the sequence of interest was confirmed by amplifying 5 ul of the phage-containing solution using qPCR conditions described below with primers cotJC F2 and cotJC R2^11^. Recombinant plaques were amplified in host cells and recovered in SM buffer as directed by manufacturer.

### Sputum sample processing and MTB specific capture DNA extraction

Mycobacterial DNA was purified from sputum specimens using a sequence specific capture illustrated in Fig. 1. Two tubes of dried reagents per reaction were prepared prior to DNA extraction as described previously^11^: one for sputum thinning and the second for specific capture probe (oligo) binding. Thinning buffer for study 1 was prepared such that 1% SDS (Sigma Aldrich), 30 mM Tris pH 8.0 (Thermo Scientific), and 10 mM EDTA (Ambion, Waltham MA) will be present after dissolution in 1 ml of sputum. For study 2, SDS was increased to 2% and EDTA was eliminated. Capture probe binding buffer for study 1 was prepared such that in 1 ml of sputum, there would be present 500 mM NaCl, 10 mM Tris pH 8.0, 1 mM EDTA, and 0.005% Tween-20 (Thermo Fisher, Waltham MA). For study 2, final composition of binding buffer was 300 mM NaCl, 60 mM MgCl2 (Sigma Aldrich), 10 mM Tris pH 8.0, and 0.005% Tween-20. All reagent tubes were dried on their side overnight in a 55 °C oven. After drying, the tubes were capped and stored at room temperature in aluminum moisture-barrier pouches (Ted Pella; Redding, CA, USA), with silica gel desiccant (McMaster Carr; Elmhurst, IL, USA) to maintain dryness and a humidity indicator card (Static Control Components; San Diego, CA, USA) to monitor moisture.

A cocktail of biotin-labeled capture oligonucleotides was prepared in advance in which a 5 µl addition contained a total of 5.5 pmol capture probe: 2.5 pmol of capture probes targeting IS*6110*, 2.5 pmol targeting the *senX3-regX3* region, and 0.5 pmol targeting *cotJC*. All capture probes were designed to target specific sequences within 100 base pairs of the amplicon. Capture probes contained a 5′ biotin moiety, included a spacer of 5 adenine residues prior to specific sequences, and were HPLC-purified. Probes were obtained from Integrated DNA Technologies (IDT, were diluted in 10 mM Tris pH 8.0, and stored at 20 °C until time of use. Proteinase K solution was prepared by combining 30 U Proteinase K (Life Technologies, Carlsbad CA, USA) in 50 ul with 1 ul 1 M CaCl2 (Sigma Aldrich).

For study 1, *Bacillus atrophaeus* spore stock (10^4^ CFU/µl; MesaLabs) was vortexed and diluted to 10^2^ CFU/µl in 40% ethanol prior to use. For study 2, lambda phage expressing the *B. atrophaeus* gene *cotJC* was diluted to 10^2^ copies/µl in TTG buffer [10 mM tris pH8, 0.01% tween-20, 10% glycerol (Affymetrix, Santa Clara, CA, USA)]. Dynabeads M-270 Streptavidin paramagnetic particles (PMPs) (Life Technologies) sufficient for 20 µl per sample were washed two times in 1 ml TT buffer (10 mM tris pH8, 0.01% tween-20) and resuspended in their initial volume of TT buffer.

All clinical sputum sample processing steps prior to heat-killing of bacteria were performed in a biological safety cabinet. Positive and negative extraction controls were prepared by adding 950 µl 10 mM Tris pH8 (study 1) or 950 µl synthetic sputum (study 2) to thinning buffer tubes, with 50,000 copies of H37Rv genomic DNA added to positive extraction control tubes only. 950 µl of sputum samples were transferred into thinning buffer tubes. In cases where the specimen was not sufficient, the volume was brought to 950 µl with ultrapure water to equalize volume across the panel. Fifty-one µl of prepared Proteinase K solution and the process control were added to each sample, vortexed, and incubated for 8 minutes at 55 °C with 1500 rpm shaking in a Benchmark Multi-Therm heater-shaker (Benchmark Scientific, Edison, NJ, USA). After 3 minutes, all samples were briefly vortexed to ensure complete mixing. The heater-shaker was then set to 100 °C with 1500 rpm shaking, and samples were incubated for 10 minutes after target temperature was reached. Next, the entire volume of thinned sample was transferred to tubes containing dried binding buffer reagents. 5 µl of capture probe cocktail were added to each sample. Samples were vortexed and incubated at 60 °C for 20 minutes (study 1) or 10 minutes (study 2) with 1500 rpm shaking^11^.

After the 60 °C incubation, 20 µl of washed PMPs were added to each sample, and samples were subjected to end-over-end rotation for 10 minutes at room temperature. Samples were then placed on a magnetic stand where PMPs were collected, and the supernatant was discarded. PMPs were washed in 1 ml TT and transferred to a clean tube. PMPs were collected and washed again in 1 ml TT, for a total of two washes. All remaining wash solution was carefully removed. Finally 11.75 µl of freshly-prepared TTG was used to resuspend test samples, and 20 µl was used for positive control samples. Samples were then eluted at 75 °C for 3 minutes with 1500 rpm shaking. PMPs were pelleted on a magnetic stand, and eluted DNA was transferred to a clean tube.

### PCR Amplification

Two MTB qPCR targets, IS*6110* and *senX3-regX3* were amplified as described in Reed, *et al*.^11^ with modifications to oligonucleotide sequences to optimize T_m_ and concentration. In lieu of primer sequences, the clarified MIQE guidelines^30^ allow publication of the reference sequence, anchor nucleotide (defined as a nucleotide located in the probe sequence), and amplicon length for each assay: IS*6110*: X17348.1, 975, 174; *senX3-regX3*: AL123456.3, 580,840, 135; and *cotJC*: CP011802.1, 497,980, 85. 2.0 mM MgCl_2_ (Sigma Aldrich) was used. PCR mixes were prepared for a 25 µl total reaction volume (15 µl master mix and 10 µl of eluted DNA).

TTG buffer was used to prepare: a standard curve of H37Rv TMC 303 genomic DNA (10^5^–10–1 copies/µl); 1:10 dilutions of positive extraction controls; and no template controls. 1000 copies of *B. atrophaeus* gBlock DNA (study 1) or lambda phage (study 2) were added as PCR positive controls for the cotJC assay. Standard curves were amplified in triplicate, and eluate from test specimens and 10X dilutions were amplified singly or in duplicate, respectively. The reactions were amplified as described previously^11^, and copy number for the MTB targets relative to the standard curve was determined by using the Qiagen Rotor-Gene Q Series Software package. PCR products were frozen at −20 °C in case of discrepant results. Samples that tested negative for both the MTB assays and had a failed PRC were considered invalid. Samples that tested positive for one or both of the MTB assays with a failed PRC were considered positive.

Sanger sequencing was used to resolve discrepant samples. The Qiagen MinElute PCR Purification kit was used to purify the entire multiplexed PCR reaction, according to manufacturer’s recommendations. DNA concentration was determined spectrophotometrically. PCR products were sequenced at the Northwestern University Center for Genomic Medicine Sequencing Core. DNA sequences were submitted to the NCBI BLAST website (https://blast.ncbi.nlm.nih.gov/Blast.cgi) for assessment of sequence identity and homology to known MTB species.

### LOD and reproducibility study

Samples for the LOD study were contrived by spiking 20, 10, or 5 cfu/ml of MTB H37Ra bacilli into a cocktail of sputum specimens prepared by vortexing 20 1 ml residual sputum specimens with approximately 7.5 g acid-washed 5 mm glass beads (Sigma Aldrich) for 5 min. 20 replicates of each concentration were evaluated following above specific capture protocol, with positive and negative controls included as described above. Samples for the reproducibility study were contrived by spiking 5000 cfu MTB H37Ra bacilli into 15 replicates of total 1 ml aliquots of three individual sputum specimens (4–6 replicates of each specimen were assayed depending on sample volume available).

### Bacillus lysis study

All DNA dilutions were prepared and all samples eluted in BD GeneOhm (BD GeneOhm™ Lysis Kit; Franklin Lakes, NJ, USA) sample buffer. A standard curve containing serial dilutions of *cotJC* gBlock® gene fragment (1e6-1e1 copies/rxn) was run as positive PCR control. No template controls and specific capture negative controls were performed, and no amplification was observed. Efficacy of the *cotJC* capture probes was demonstrated by extracting and amplifying 10,000 copies *cotJC* gBlock using the standard protocol. Prior to treatments described below, spores were centrifuged for 10 minutes at 14,000 rpm in an Eppendorf 5415c centrifuge (Hauppauge, NY, USA), and supernatant was removed. For each treatment, 10,000 spores were nominally present in 10 µl final volume, to be added to a 25 µl total PCR reaction.

To test for lysis of spores by heat, BD GeneOhm sample buffer was added to collected spores to a total volume of 15~20 µl. Samples were heated for 10 minutes at 95 °C with shaking (1500 rpm). To assess lysis of spores by the GeneOhm kit, collected spores were resuspended in 50 µl BD GeneOhm sample buffer, transferred to a GeneOhm lysis tube, vortexed for 5 minutes at maximum speed (3000 rpm), and centrifuged gently to collect the liquid. The GeneOhm protocol called for samples to be incubated at 95 °C for 2 minutes with shaking (1500 rpm). To evaluate specific capture of GeneOhm-lysed spores, collected spores were resuspended and processed in GeneOhm lysis tubes as above. Entire volume was transferred to a tube containing dried thinning buffer. Water was added to 950 µl, and samples were extracted as above. To evaluate specific capture of untreated spores, collected spores were resuspended in 950 µl water, which was transferred to a tube of thinning buffer and extracted as above.

### Statistical Methods

C_q_ values were plotted against log (copy number) using Qiagen’s RGQ software to obtain standard curves. Slope parameters were estimated by linear regression of C_q_ values vs log copy number; the 95% Confidence Intervals of slope estimates were calculated in XLStat. Test sensitivity, confidence intervals and two-tailed student t tests were calculated using Vassar Stats online statistical package (www.vassarstats.net/index.html). P values were calculated from t values and degrees of freedom using Free Statistics Calculator (http://www.danielsoper.com/statcalc/calculator.aspx?id=8). PCR Efficiency was calculated from the slope of the standard curve, Eff% = −1 + 10^(−1/slope)^. Box and whisker plots were made using BoxPlotR (http://shiny.chemgrid.org/boxplotr/).

## Electronic supplementary material

supplementary tables

## References

[1] World Health Organization. Global Tuberculosis Report 2016. Available at: http://www.who.int/tb/publications/global_report/en/ (Accessed on: 10^th^ November 2016).

[2] Bates M, Zumla A (2016). The development, evaluation and performance of molecular diagnostics for detection of *Mycobacterium tuberculosis*. Expert Rev Mol Diagn..

[3] Dowdy DW, Chaisson RE, Maartens G, Corbett EL, Dorman SE (2008). Impact of enhanced tuberculosis diagnosis in South Africa: a mathematical model of expanded culture and drug susceptibility testing. Proceedings of the National Academy of Sciences of the United States of America..

[4] O’Connor JA, O’Reilly B, Corcoran GD, O’Mahony J, Lucey B (2015). Mycobacterium diagnostics: from the primitive to the promising. British Journal of Biomedical Science..

[5] van Zyl-Smit RN (2011). Comparison of Quantitative Techniques including Xpert MTB/RIF to Evaluate Mycobacterial Burden. PLoS ONE..

[6] ATS/CDC/IDSA (2000). Diagnostic standards and classification of tuberculosis in adults and children. Am J Respir Crit Care Med..

[7] Steingart KR (2014). Xpert(R) MTB/RIF assay for pulmonary tuberculosis and rifampicin resistance in adults. Cochrane Database Syst. Rev..

[8] Theron G (2014). Feasibility, accuracy, and clinical effect of point-of-care Xpert MTB/RIF testing for tuberculosis in primary-care settings in Africa: a multicentre, randomised, controlled trial. Lancet..

[9] Denkinger CM (2015). Defining the needs for next generation assays for tuberculosis. The Journal of Infectious Diseases..

[10] Mangiapan G (1996). Sequence capture-PCR improves detection of mycobacterial DNA in clinical specimens. Journal of Clinical Microbiology..

[11] Reed JL (2016). Highly sensitive sequence specific qPCR detection of *Mycobacterium tuberculosis* complex in respiratory specimens. Tuberculosis..

[12] Lok KH (2002). Molecular differentiation of *Mycobacterium tuberculosis* strains without IS*6110* insertions. Emerging Infectious Diseases..

[13] Thierry D (1990). Characterization of a *Mycobacterium tuberculosis* insertion sequence, IS*6110*, and its application in diagnosis. J Clin Microbiol..

[14] Parish T, Smith DA, Roberts G, Betts J, Stoker NG (2003). The *senX3-regX3* two-component regulatory system of *Mycobacterium tuberculosis* is required for virulence. Microbiology..

[15] Helb D (2010). Rapid detection of *Mycobacterium tuberculosis* and rifampin resistance by use of on-demand, near-patient technology. J Clin Microbiol..

[16] Zheng H (2008). Genetic basis of virulence attenuation revealed by comparative genomic analysis of *Mycobacterium tuberculosis* strain H37Ra versus H37Rv. PLoS ONE..

[17] Blakemore R (2011). A Multisite assessment of the quantitative capabilities of the Xpert MTB/RIF assay. American Journal of Respiratory and Critical Care Medicine..

[18] Belgrader P (1999). A minisonicator to rapidly disrupt bacterial spores for DNA analysis. Analytical Chemistry..

[19] Stocher M, Berg J (2004). Internal control DNA for PCR assays introduced into lambda phage particles exhibits nuclease resistance. Clinical Chemistry..

[20] Shawar RM, el-Zaatari FA, Nataraj A, Clarridge JE (1993). Detection *of Mycobacterium tuberculosis* in clinical samples by two-step polymerase chain reaction and nonisotopic hybridization methods. J Clin Microbiol..

[21] Theron G (2014). Determinants of PCR performance (Xpert MTB/RIF), including bacterial load and inhibition, for TB diagnosis using specimens from different body compartments. Scientific Reports..

[22] Huyen MN (2013). Characterisation of *Mycobacterium tuberculosis* isolates lacking IS*6110* in Viet Nam. Int J Tuberc Lung Dis..

[23] Caliendo AM (2013). Better tests, better care: improved diagnostics for infectious diseases. Clinical Infectious Diseases..

[24] Alland, D. *et al*. Xpert MTB/RIF Ultra: a new near-patient TB test with sensitivity equal to culture. CROI 2015; Boston, MA. Available at: http://www.croiconference.org/sessions/xpert-mtbrif-ultra-new-near-patient-tb-test-sensitivity-equal-culture. (Accessed: 24^th^ March 2017).

[25] Pang Y (2016). Factors associated with missed detection of *Mycobacterium tuberculosis* by automated BACTEC MGIT 960 system. BioMed Research International..

[26] Theron G (2016). Xpert MTB/RIF results in patients with previous tuberculosis: can we distinguish true from false positive results?. Clinical Infectious Diseases..

[27] Devonshire AS (2016). The use of digital PCR to improve the application of quantitative molecular diagnostic methods for tuberculosis. BMC Infectious Diseases..

[28] Aziz, M. External quality assessment for AFB smear microscopy. Available at: https://stacks.cdc.gov/view/cdc/11440 (Accessed on: 20^th^ March 2017). (2002).

[29] Du Y (2015). A Sweet spot for molecular diagnostics: coupling isothermal amplification and strand exchange circuits to glucometers. Scientific Reports..

[30] Bustin SA (2011). Primer sequence disclosure: a clarification of the MIQE guidelines. Clinical Chemistry..

