# Supplementary material for: XtracTB Assay, a Mycobacterium tuberculosis molecular screening test with sensitivity approaching culture
Source: Sci Rep. 2017 Jun 16;7:3653. doi: 10.1038/s41598-017-03930-3 (PMC5473816; doi:10.1038/s41598-017-03930-3)
Supplement: Supplementary file 1 — supplementary tables [file 41598_2017_3930_MOESM1_ESM.pdf]

XtracTB Assay, a *Mycobacterium tuberculosis* molecular screening test with sensitivity approaching culture

Jennifer L. Reed, Debby Basu, Matthew A. Butzler, & Sally M. McFall

Supplemental Table 1. Summary of XtracTB Assay results in study of specimens supplied by FIND. SSM-/C+ = sputum smear negative/culture positive; VN = Vietnam and ZA = South Africa. SSM+/C+= sputum smear positive/culture positive; SCA/C+= scanty sputum smear positive/culture positive; CXR+/C-= chest X-ray positive/culture negative; and C-= culture negative. + = positive; - = negative, I = invalid, LVI = low volume invalid, less than 500 µl sputum tested and result was negative.

**Extract. No**	**FIND ID**	**Country of Origin**	**Sputum Vol**	**FIND Result**	**IS*6110* qPCR**	***senX3-regX3* copy no**	**PRC-*cotJC* qPCR**	**XtracTB Result**
1	4137	PERU	950	SSM-/C+	+	4.4E+1	+	+
2	4266	PERU	950	SSM-/C+	+	>1.0E+5	+	+
3	4270	PERU	950	SSM-/C+	+	4.4E+1	-	+
4	4288	PERU	950	SSM-/C+	+	>1.0E+5	+	+
5	4305	PERU	850	SSM-/C+	+	-	-	+
6	4330	PERU	950	SSM-/C+	+	6.8E+1	-	+
7	4332	PERU	950	SSM-/C+	+	4.9E+4	+	+
8	4334	PERU	950	SSM-/C+	+	<10	-	+
9	4338	PERU	950	SSM-/C+	+	3.0E+4	+	+
10	4348	PERU	950	SCA/C+	+	>1.0E+5	+	+
11	4349	PERU	950	SSM-/C+	+	1.2E+4	+	+
12	4421	PERU	950	SSM-/C+	+	>1.0E+5	+	+
13	4431	PERU	950	SSM-/C+	+	6.0E+2	+	+
14	4456	PERU	950	SSM-/C+	+	>1.0E+5	+	+
15	4458	PERU	950	SSM-/C+	+	1.9E+3	-	+
16	4518	PERU	950	SSM-/C+	+	4.1E+1	+	+
17	4526	PERU	950	SSM-/C+	+	<10	+	+
18	4527	PERU	950	SSM-/C+	-	-	-	I
19	4533	PERU	950	SSM-/C+	+	8.0E+2	+	+
20	4538	PERU	950	SSM-/C+	+	1.4E+1	+	+

Extract. No	FIND ID	Country of Origin	Sputum Vol	FIND Result	IS*6110* qPCR	*senX3-regX3* copy no	PRC-*cotJC* qPCR	XtracTB Result
21	4543	PERU	950	SSM-/C+	+	<10	+	+
22	4544	PERU	950	SSM-/C+	+	<10	-	+
23	4549	PERU	950	SSM-/C+	+	-	-	+
24	4558	PERU	950	SSM+/C+	+	>1.0E5	+	+
25	4586	PERU	950	SSM-/C+	+	4.4E1	+	+
26	4625	PERU	950	SSM-/C+	+	6.6E3	+	+
27	4631	PERU	950	SSM-/C+	+	7.3E3	+	+
28	4697	PERU	950	SCA/C+	+	1.4E3	-	+
29	4708	PERU	950	SCA/C+	+	2.3E4	+	+
30	4737	PERU	950	SCA/C+	+	9.5E2	+	+
31	4739	PERU	850	SSM-/C+	+	9.5E1	-	+
32	4765	PERU	750	SSM-/C+	+	6.7E1	+	+
33	4769	PERU	950	SSM-/C+	+	2.8E1	+	+
34	4845	PERU	900	SSM+/C+	+	2.9E4	+	+
35	4862	PERU	950	SSM+/C+	+	>1.0E5	-	+
36	4932	PERU	950	SSM+/C+	+	>1.0E5	+	+
37	4934	PERU	950	SSM+/C+	+	1.1E4	+	+
38	4953	PERU	950	SSM+/C+	+	9.0E4	+	+
39	4956	PERU	950	SSM+/C+	+	8.4E3	-	+
40	4959	PERU	950	SSM+/C+	+	>1.0E5	-	+
41	4963	PERU	950	SSM+/C+	+	>1.0E5	+	+
42	4965	PERU	950	SSM+/C+	+	>1.0E5	-	+
43	4966	PERU	950	SSM+/C+	+	>1.0E5	+	+

Extract. No	FIND ID	Country of Origin	Sputum Vol	FIND Result	IS*6110* qPCR	*senX3-regX3* copy no	PRC-*cotJC* qPCR	XtracTB Result
44	4982	PERU	950	SSM+/C+	+	2.1E2	+	+
45	4985	PERU	950	SSM+/C+	+	>1.0E5	+	+
46	4987	PERU	950	SSM+/C+	+	>1.0E5	+	+
47	4988	PERU	950	SSM+/C+	+	5.2e4	+	+
48	5003	PERU	950	SSM+/C+	+	3.0E4	+	+
49	5011	PERU	750	SSM+/C+	+	2.2E4	-	+
50	5013	PERU	700	SSM+/C+	+	2.6E4	+	+
51	5042	PERU	950	SSM+/C+	+	>1.0E5	-	+
52	5043	PERU	950	SSM+/C+	+	8.6E3	+	+
53	93	VN	950	SCA/C+	+	7.6E2	-	+
54	182	VN	950	SCA/C+	+	1.6E4	+	+
55	247	VN	950	SCA/C+	-	-	-	I
56	310	VN	950	SCA/C+	+	6.7E3	+	+
57	343	VN	950	SCA/C+	+	2.8E3	+	+
58	352	VN	950	SCA/C+	+	7.1E3	+	+
59	390	VN	950	SCA/C+	+	3.5E3	+	+
60	408	VN	950	SCA/C+	+	1.4E2	+	+
61	456	VN	850	SCA/C+	+	1.2E3	+	+
62	464	VN	950	SSM-/C+	+	<10	-	+
63	475	VN	850	SCA/C+	+	1.9E3	+	+
64	520	VN	950	SCA/C+	+	-	+	+
65	550	VN	950	SCA/C+	+	<10	+	+
66	678	VN	950	SCA/C+	+	7.6E4	+	+

Extract. No	FIND ID	Country of Origin	Sputum Vol	FIND Result	IS*6110* qPCR	*senX3-regX3* copy no	PRC-*cotJC* qPCR	XtracTB Result
67	788	VN	950	SSM-/C+	+	6.8E3	+	+
68	797	VN	950	SCA/C+	+	5.3E2	+	+
69	859	VN	950	SCA/C+	+	2.3E1	-	+
70	983	VN	950	SCA/C+	+	2.0E4	+	+
71	2015	VN	850	CXR+/C-	-	-	-	I
72	2027	VN	900	CXR+/C-	-	-	+	-
73	2037	VN	950	CXR+/C-	-	-	+	-
74	2042	VN	950	CXR+/C-	-	<10	+	+
75	57	ZA	900	C-	+	1.2E4	-	+
76	85	ZA	700	C-	-	-	+	-
77	86	ZA	900	C-	-	-	+	-
78	91	ZA	950	C-	-	-	+	-
79	96	ZA	450	C-	-	-	+	LVI
80	125	ZA	250	C-	-	-	+	LVI
81	133	ZA	250	C-	-	-	+	LVI
82	160	ZA	450	C-	-	-	+	LVI
83	174	ZA	900	C-	-	-	+	-
84	175	ZA	350	C-	-	-	+	LVI
85	178	ZA	450	C-	-	-	+	LVI
86	194	ZA	300	C-	-	-	+	LVI
87	199	ZA	400	C-	-	-	+	LVI
88	206	ZA	300	C-	-	-	+	LVI
89	211	ZA	300	C-	+	<10	+	+

**Extract. No**	**FIND ID**	**Country of Origin**	**Sputum Vol**	**FIND Result**	**IS*6110* qPCR**	***senX3-regX3* copy no**	**PRC-*cotJC* qPCR**	**XtracTB Result**
90	215	ZA	350	C-	-	-	+	LVI
91	219	ZA	300	C-	-	-	+	LVI
92	227	ZA	400	C-	-	-	+	LVI
93	190	ZA	300	C-	-	-	+	LVI
94	231	ZA	300	C-	-	-	+	LVI

Table 2. Summary of FIND specimen volumes. SSM- = sputum smear microscopy negative; SCA = sputum smear microscopy scanty positive; SSM+ = sputum smear microscopy positive

Category	N	No. with Sufficient Sample (950 µl)	No. with Reduced Vol. Tested; Vol. Range
**SSM+**	20	17 (85%)	3 (15%); 700-900 µl
**SCA**	20	18 (90%)	2 (10%); 850 µl
**SSM-**	30	28 (93%)	2 (7%); 750-850 µl
**Culture-**	20	1 (5%)	12 (60%); 200-400 µl<br>7 (35%); 401-900 µl
**CXR**	4	2 (50%)	2 (50%);850-900 µl

Supplemental Table 3. Summary of XtracTB Assay results in study of specimens supplied by FIND. VN = Vietnam and ZA = South Africa. SSM-/C+ = sputum smear negative/culture positive; SSM+/C+= sputum smear positive/culture positive; SSM-/C- = sputum smear negative/culture negative. + = positive, - = negative, I = invalid. LFU = lost to follow up, sample excluded by FIND.

**Extract. No**	**FIND ID**	**Country of Origin**	**Sputum Vol**	**FIND Result**	**IS*6110* qPCR**	***senX3-regX3* cn / sample**	**PRC-*cotJC* qPCR**	**XtracTB Result**
1	01 18 0088 Sp1 01	ZA	600	SSM+/C+	-	-	+	-
2	01 18 0090 Sp1 03	ZA	650	SSM+/C+	+	5.7E+03	+	+
3	01 18 0096 Sp1 02	ZA	950	LFU	+	1.9E+05	+	+
4	01 18 0097 Sp1 02	ZA	950	SSM-/C+	+	1.1E+02	+	+
5	01 41 0127 Sp1 02	ZA	950	C-	-	-	+	-
6	01 14 2083 Sp1 01	VN	800	SSM-/C+	-	-	+	-
7	01 14 2085 Sp1 01	VN	950	SSM-/C+	+	4.5E+02	+	+
8	01 14 2087 Sp1 01	VN	950	SSM-/C+	-	-	+	-
9	01 14 2090 Sp1 01	VN	800	SSM-/C+	+	<10	+	+
10	01 14 2093 Sp1 01	VN	950	SSM-/C+	+	3.5E+01	+	+
11	01 14 2105 Sp2 01	VN	950	SSM-/C+	-	-	+	-
12	01 14 2108 Sp1 01	VN	950	SSM-/C+	+	1.2E+01	+	+
13	01 14 2114 Sp2 01	VN	950	SSM-/C+	-	-	-	I
14	01 14 2122 Sp1 01	VN	950	SSM-/C+	+	7.0E+02	+	+
15	01 14 2123 Sp1 01	VN	950	SSM-/C+	+	3.6E+01	+	+
16	01 14 2129 Sp1 01	VN	950	SSM-/C+	+	-	+	+
17	01 14 2130 Sp2 01	VN	950	SSM-/C+	+	3.1E+03	+	+
18	01 14 2141 Sp1 01	VN	600	SSM-/C+	+	<10	+	+
19	01 14 2147 Sp1 01	VN	600	SSM-/C+	+	<10	+	+
20	01 14 2152 Sp2 01	VN	950	SSM-/C+	+	1.5E+01	+	+

21	01 01 4178 Sp1 03	PERU	950	C-	-	-	-	I
22	01 01 4180 Sp1 03	PERU	950	C-	-	-	+	-
23	01 01 4188 Sp1 03	PERU	950	C-	-	-	+	-
24	01 01 4192 Sp1 01	PERU	950	C-	-	-	+	-
25	01 01 4194 Sp1 03	PERU	950	C-	-	-	+	-
26	01 01 4199 Sp1 01	PERU	950	C-	-	-	+	-
27	01 01 4220 Sp1 03	PERU	950	C-	-	-	+	-
28	01 01 4226 Sp1 03	PERU	950	C-	-	-	+	-
29	01 01 4229 Sp1 03	PERU	700	C-	-	-	+	-
30	01 01 4238 Sp1 05	PERU	950	C-	-	-	+	-
31	01 01 4240 Sp1 04	PERU	950	C-	-	-	+	-
32	01 01 4262 Sp1 03	PERU	950	C-	-	-	+	-
33	01 01 4272 Sp1 01	PERU	950	C-	-	-	+	-
34	01 01 4280 Sp1 03	PERU	950	C-	-	-	+	-
35	01 01 4288 Sp2 01	PERU	950	SSM-/C+	+	7.2E+04	+	+
36	01 01 4302 Sp1 02	PERU	950	C+	+	7.4E+03	+	+
37	01 01 4309 Sp1 01	PERU	950	SSM+/C+	+	3.5E+02	-	+
38	01 01 4314 Sp1 04	PERU	950	SSM+/C+	+	1.8E+01	-	+
39	01 01 4315 Sp1 04	PERU	950	SSM+/C+	+	7.0E+01	+	+
40	01 01 4323 Sp2 04	PERU	950	SSM-/C+	+	<10	-	+
41	01 01 4331 Sp1 02	PERU	950	SSM+/C+	+	5.1E+01	+	+
42	01 01 4333 Sp2 02	PERU	950	SSM+/C+	+	<10	-	+
43	01 01 4335 Sp1 01	PERU	950	SSM+/C+	+	<10	-	+
44	01 01 4346 Sp1 05	PERU	950	SSM+/C+	+	8.6E+03	+	+
45	01 01 4363 Sp1 01	PERU	950	C-	-	-	+	-
46	01 01 4368 Sp1 05	PERU	950	C-	-	-	-	I

47	01 01 4381 Sp1 01	PERU	950	C-	-	-	+	-
48	01 01 4384 Sp1 02	PERU	950	C-	-	-	+	-
49	01 01 4405 Sp2 01	PERU	950	SSM+/C+	+	3.9E+01	+	+
50	01 01 4419 Sp2 04	PERU	950	SSM+/C+	+	4.2E+03	+	+
51	01 01 4433 Sp2 01	PERU	950	SSM+/C+	+	1.4E+03	+	+
52	01 01 4434 Sp2 01	PERU	950	SSM-/C+	+	<10	+	+
53	01 01 4439 Sp1 03	PERU	500	C-	-	-	+	-
54	01 01 4449 Sp2 04	PERU	950	SSM-/C+	+	<10	-	+
55	01 01 4450 Sp1 02	PERU	950	C-	-	-	+	-
56	01 01 4456 Sp1 02	PERU	950	SSM-/C+	+	5.4E+02	-	+
57	01 01 4457 Sp1 03	PERU	950	SSM+/C+	+	2.6E+04	+	+
58	01 01 4460 Sp1 04	PERU	950	SSM-/C+	+	3.5E+01	+	+
59	01 01 4470 Sp1 02	PERU	950	C-	-	-	+	-
60	01 01 4471 Sp1 04	PERU	950	C-	-	-	+	-
61	01 01 4473 Sp1 03	PERU	950	C-	-	-	+	-
62	01 01 4474 Sp1 02	PERU	950	SSM-/C+	+	4.2E+05	+	+
63	01 01 4475 Sp1 03	PERU	950	C-	-	-	+	-
64	01 01 4480 Sp2 04	PERU	950	SSM-/C+	+	5.2E+02	-	+
65	01 01 4482 Sp2 02	PERU	950	SSM-/C+	+	1.3E+05	+	+
66	01 01 4486 Sp1 01	PERU	950	C-	-	-	+	-
67	01 01 4488 Sp1 03	PERU	950	C-	-	-	+	-
68	01 01 4491 Sp1 02	PERU	950	C-	-	-	+	-
69	01 01 4494 Sp1 04	PERU	950	C-	-	-	+	-
70	01 01 4495 Sp1 04	PERU	950	C-	-	-	+	-
71	01 01 4496 Sp1 01	PERU	950	C-	-	-	+	-
72	01 01 4497 Sp2 05	PERU	950	SSM-/C+	+	8.4E+03	+	+

73	01 01 4500 Sp1 02	PERU	950	C-	-	-	-	I
74	01 01 4507 Sp1 01	PERU	950	C-	-	-	+	-
75	01 01 4508 Sp1 03	PERU	950	C-	-	-	+	-
76	01 01 4509 Sp1 04	PERU	950	C-	-	-	+	-
77	01 01 4565 Sp1 03	PERU	950	C-	-	-	+	-
78	01 01 4585 Sp2 01	PERU	950	SSM-/C+	+	4.4E+01	+	+
79	01 01 4587 Sp2 04	PERU	950	SSM-/C+	+	1.2E+01	-	+
80	01 01 4592 Sp1 04	PERU	950	C-	-	-	+	-
81	01 01 4596 Sp1 02	PERU	950	C-	-	-	+	-
82	01 01 4597 Sp1 01	PERU	950	C-	-	-	+	-
83	01 01 4603 Sp1 01	PERU	950	SSM+/C+	+	1.9E+02	+	+
84	01 01 4604 Sp1 01	PERU	950	SSM-/C+	+	4.3E+02	+	+
85	01 01 4605 Sp1 01	PERU	950	SSM-/C+	+	6.0E+01	+	+
86	01 01 4627 Sp1 02	PERU	950	SSM+/C+	+	2.1E+05	+	+
87	01 01 4628 Sp1 03	PERU	950	SSM+/C+	+	>1E6	+	+
88	01 01 4629 Sp1 01	PERU	350	SSM-/C+	+	3.5E+00	+	+
89	01 01 4636 Sp1 04	PERU	950	C-	-	-	+	-
90	01 01 4648 Sp1 03	PERU	950	C-	-	-	+	-
91	01 01 4653 Sp2 04	PERU	950	SSM-/C+	+	<10	+	+
92	01 01 4657 Sp1 03	PERU	950	SSM+/C+	+	1.4E+04	+	+
93	01 01 4663 Sp1 04	PERU	950	C-	-	-	+	-
94	01 01 4667 Sp1 04	PERU	950	C-	-	-	-	I
95	01 01 4668 Sp1 03	PERU	950	C-	-	-	+	-
96	01 01 4674 Sp1 02	PERU	950	SSM+/C+	+	6.7E+04	+	+
97	01 01 4683 Sp1 01	PERU	900	SSM+/C+	+	<10	+	+
98	01 01 4684 Sp1 02	PERU	950	SSM+/C+	+	1.3E+05	+	+

99	01 01 4685 Sp1 03	PERU	950	SSM+/C+	+	>1E6	+	+
100	01 01 4687 Sp1 01	PERU	950	SSM+/C+	+	2.7E+03	+	+
101	01 01 4688 Sp1 01	PERU	950	SSM+/C+	+	2.9E+04	+	+
102	01 01 4695 Sp1 01	PERU	950	SSM+/C+	+	8.2E+01	+	+
103	01 01 4696 Sp1 01	PERU	950	SSM+/C+	+	1.3E+04	+	+
104	01 01 4697 Sp1 04	PERU	950	SSM+/C+	+	1.6E+03	+	+
105	01 01 4700 Sp1 01	PERU	950	SSM+/C+	+	>1E6	+	+
106	01 01 4701 Sp1 03	PERU	950	SSM+/C+	+	1.7E+05	+	+
107	01 01 4703 Sp1 03	PERU	950	SSM+/C+	+	<10	+	+
108	01 01 4704 Sp1 02	PERU	950	SSM+/C+	+	6.4E+05	+	+
109	01 01 4705 Sp1 03	PERU	800	SSM+/C+	+	9.7E+04	+	+
110	01 01 4706 Sp1 02	PERU	950	SSM+/C+	+	4.8E+05	+	+
111	01 01 4710 Sp1 02	PERU	950	SSM+/C+	+	2.5E+04	+	+
112	01 01 4713 Sp2 04	PERU	950	SSM-/C+	-	-	+	-
113	01 01 4715 Sp1 02	PERU	950	SSM+/C+	+	>1E6	+	+
114	01 01 4720 Sp1 02	PERU	950	SSM+/C+	+	2.5E+03	+	+
115	01 01 4723 Sp1 03	PERU	950	SSM+/C+	+	1.6E+05	+	+
116	01 01 4724 Sp1 02	PERU	950	SSM+/C+	+	7.2E+05	+	+
117	01 01 4725 Sp1 03	PERU	950	SSM+/C+	+	<10	+	+
118	01 01 4726 Sp1 01	PERU	950	SSM+/C+	+	7.0E+05	+	+
119	01 01 4728 Sp1 02	PERU	950	SSM+/C+	+	<10	+	+
120	01 01 4743 Sp1 03	PERU	950	C-	-	-	+	-
121	01 01 4750 Sp1 04	PERU	950	SSM+/C+	+	1.1E+01	-	+
122	01 01 4761 Sp2 04	PERU	950	SSM-/C+	+	8.7E+01	+	+
123	01 01 4765 Sp2 03	PERU	750	SSM-/C+	+	1.5E+02	+	+
124	01 01 4782 Sp2 01	PERU	800	SSM-/C+	+	-	+	+

125	01 01 4787 Sp1 01	PERU	800	SSM+/C+	+	<10	+	+
126	01 01 4793 Sp1 02	PERU	950	C-	-	-	-	I
127	01 01 4795 Sp1 04	PERU	800	SSM+/C+	+	-	+	+
128	01 01 4796 Sp1 01	PERU	950	SSM+/C+	+	1.1E+01	-	+
129	01 01 4800 Sp1 02	PERU	500	SSM+/C+	+	2.7E+04	+	+
130	01 01 4802 Sp1 03	PERU	950	SSM+/C+	+	1.2E+02	+	+
131	01 01 4822 Sp1 05	PERU	950	SSM+/C+	+	4.7E+03	+	+
132	01 01 4823 Sp1 01	PERU	950	SSM+/C+	+	2.3E+04	+	+
133	01 01 4824 Sp1 03	PERU	800	SSM+/C+	+	3.2E+05	+	+
134	01 01 4826 Sp1 03	PERU	950	SSM+/C+	+	1.3E+02	-	+
135	01 01 4827 Sp1 03	PERU	950	SSM+/C+	+	<10	+	+
136	01 01 4828 Sp1 02	PERU	950	SSM+/C+	+	3.9E+03	+	+
137	01 01 4865 Sp1 03	PERU	950	SSM+/C+	+	3.7E+04	+	+
138	01 01 4868 Sp1 03	PERU	950	SSM+/C+	+	3.6E+01	+	+
139	01 01 4886 Sp1 02	PERU	750	SSM+/C+	+	3.6E+05	+	+
140	01 01 4889 Sp1 01	PERU	950	SSM+/C+	-	-	-	I
141	01 01 4894 Sp1 02	PERU	950	SSM+/C+	+	6.2E+03	+	+
142	01 01 4895 Sp1 03	PERU	950	SSM+/C+	+	3.4E+02	+	+
143	01 01 4897 Sp1 03	PERU	950	SSM+/C+	+	1.5E+05	+	+
144	01 01 4903 Sp1 03	PERU	700	C-	-	-	+	-
145	01 01 4913 Sp1 01	PERU	950	SSM+/C+	+	6.2E+02	+	+
146	01 01 4915 Sp1 02	PERU	950	SSM+/C+	+	4.5E+01	-	+
147	01 01 4949 Sp1 04	PERU	950	C-	-	-	+	-
148	01 01 4995 Sp1 04	PERU	950	C-	-	-	+	-
149	01 01 5013 Sp1 04	PERU	950	SSM+/C+	+	8.5E+03	-	+
150	01 01 5043 Sp1 01	PERU	200	SSM+/C+	+	2.2E+03	+	+
